# Compound Chinese medicine (F1) improves spleen deficiency diarrhea by protecting the intestinal mucosa and regulating the intestinal flora

**DOI:** 10.3389/fmicb.2023.1292082

**Published:** 2024-01-16

**Authors:** Kang Wang, Guanzong Li, Zhi Yang, Fumei Yang, Yulin Sun, Gang Duan, Wang Sun, Ke Zhou, Jun He, Feiyan Dai

**Affiliations:** ^1^College of Veterinary Medicine, Yunnan Agricultural University, Kunming, China; ^2^College of Veterinary Medicine, South China Agricultural University, Guangzhou, China; ^3^Animal Disease Prevention and Control Center of Chuxiong, Chuxiong, China; ^4^Veterinarian, Kunming Technical Contract Accreditation and Registration Station, Kunming, China

**Keywords:** compound Chinese medicine, SDD, intestinal mucosa, intestinal flora, regulate

## Abstract

Compound Chinese medicine (F1) is a traditional prescription in Chinese medicine that is commonly used to treat spleen deficiency diarrhea (SDD). It has demonstrated remarkable effectiveness in clinical practice. However, the precise mechanism by which it exerts its antidiarrheal effect is still unclear. This study aimed at investigating the antidiarrheal efficacy and mechanism of F1 on senna-induced secretory diarrhea (SDD). Senna was utilized to induce the development of a mouse model of senna-induced secretory diarrhea (SDD) in order to observe the rate of diarrhea, diarrhea index, blood biochemistry, and histopathological changes in the small intestine. Additionally, the levels of sodium and hydrogen exchange protein 3 (NHE3) and short-chain fatty acids (SCFAs) were determined using enzyme-linked immunosorbent assay (ELISA). The impact of F1 on the senna-induced SDD mouse models was evaluated by monitoring changes in the gut microbiota through 16S rRNA (V3-V4) sequencing. The results demonstrated that F1, a traditional Chinese medicine, effectively increased the body weight of SDD mice and reduced the incidence of diarrhea and diarrhea index. Additionally, F1 restored liver and kidney function, reduced the infiltration of inflammatory cells in intestinal tissue, and promoted the growth of intestinal villi. Furthermore, F1 was found to enhance the expression of NHE3 and SCFAs. It also increased the abundance of Firmicutes and Lactobacillus species, while decreasing the abundance of Proteobacteria and Shigella.

## 1 Introduction

Spleen deficiency diarrhea is a prevalent gastrointestinal syndrome in clinical practice (Ma et al., 2019; Shi et al., 2019). It is primarily caused by congenital endowment deficiency or prolonged illness, leading to weakness in the spleen and stomach, impaired digestion, and diarrhea (Mei et al., 2022). Some literature has pointed out that the irritable bowel syndrome caused by weak spleen and stomach is as high as 57.5% (Zhu et al., 2016). The spleen functions optimally in a dry environment. However, when the spleen and stomach are weak, they struggle to digest and metabolize food, resulting in the formation of a cold and humid environment that further exacerbates the symptoms of spleen deficiency (Su et al., 2021). The symptoms include diarrhea with loose stools, weight loss, and dull fur. Based on the pathogenesis characteristics of the deficiency of spleen yang, the treatment focuses on the principle of regulating spleen and stomach (Wu, 1998). Chinese herbal medicine, as a significant component of complementary and alternative medicine, has gained considerable acceptance in clinical treatment. It is noteworthy that data reveals that 61.6% of patients suffering from digestive diseases have sought the benefits of traditional Chinese medicine (Tan et al., 2020). In clinical practice, the commonly used treatments are Sijunzi Decoction, Shengmai Yin, Shenling Baizhu San, etc., combined with spicy or bitter Chinese medicine to invigorate the spleen and eliminate dampness (Yu et al., 2016; Gan et al., 2017; You et al., 2020; Chen et al., 2022).

According to modern medicine, the concept of the ‘spleen’ involves the functions of multiple systems such as digestion, endocrine, and immunity (Lewis et al., 2019; Willard-Mack et al., 2019; Li et al., 2021). Traditional Chinese medicine considers the intestine to be an important component of spleen function. Recent medical research has also found a close association between small intestinal bacterial overgrowth (SIBO), gastrointestinal digestive dysfunction, intestinal inflammation, decreased immune function, and intestinal flora disorders (Brown et al., 2022; Banaszak et al., 2023; Li et al., 2023). The intestinal mucosal epithelium acts as a selective barrier that protects the host from harmful substances while allowing nutrient absorption (Turner, 2009; Fa et al., 2016). Disruption of this barrier can result in increased permeability, inflammation, reduced NHE3 activity, and other pathological changes, ultimately leading to diarrhea (Peritore-Galve et al., 2023). In recent years, there have been increasing reports on the potential of traditional Chinese medicine to improve the health of the intestinal mucosa (An et al., 2022). For instance, berberine has been found to protect the intestinal mucosal barrier function by reducing intestinal macrophage infiltration and the inflammatory response (Li et al., 2020; Dong et al., 2022). Additionally, Poria polysaccharide has shown promising results in reducing intestinal mucosal injury and inflammation in mice, thereby improving intestinal barrier function (Xu et al., 2023).

The intestinal flora and the animal immune system have a mutually beneficial relationship, known as the ‘flora-host’ symbiotic relationship (Zhou et al., 2020). The active involvement of the intestinal flora and its metabolites in maintaining the balance of the intestinal immune system has a positive impact on preventing and controlling intestinal diseases, and it also plays an essential role in regulating animal health (Liu et al., 2022). When the dynamic balance of the intestinal microecosystem is disrupted, the intestinal flora becomes imbalanced, leading to various diseases both inside and outside the intestine (Hu and Yang, 2022; Liu et al., 2022). Traditional Chinese medicine has gained significant attention and research from experts and scholars due to its influence on the intestinal flora and its ability to regulate bodily functions (Che et al., 2022; Li et al., 2022). Since Chinese herbal medicines are typically consumed orally, their active ingredients directly interact with the intestinal flora, thereby influencing the structure of the intestinal flora and its metabolites. Cang Zhu Compound has been shown to regulate the structure of intestinal flora, improving the intestinal microenvironment (Ma et al., 2019). Additionally, Huo Xiang Zhengqi oral liquid has been found to promote beneficial bacteria in the intestinal flora and inhibit the growth of pathogenic bacteria, effectively alleviating symptoms of wet spleen and stomach in rats. Research has also highlighted the close relationship between traditional Chinese medicine and the regulation of intestinal flora metabolites, such as short-chain fatty acids, amino acids, bile acids, indole, and its derivatives (Feng et al., 2018; Sun et al., 2022; Li et al., 2023). Therefore, maintaining a balanced intestinal flora and regulating microbial metabolites have emerged as new avenues of study in understanding the mechanism of action of traditional Chinese medicine.

This study aims to investigate the antidiarrheal effect of F1 and explore its curative potential in the treatment of SDD. The specific mechanisms involved, including the regulation of intestinal microbiota and the maintenance of intestinal barrier function, will be elucidated. The findings of this study will provide a theoretical reference for the clinical application of F1.

## 2 Materials and methods

### 2.1 Experimental consumables

Experimental Chinese medicine pieces such as Codonopsis, Largehead Atractylodes Rhizome, Poria, Dried Ginger, Yam, Rhizome of Szechuan Lovage, Knotweed, Fortune Eupatorium Herb, Herba Lycopi, Coptis chinensis Franch, Divine Comedy, Senna, etc.

Mouse sodium hydrogen exchange factor 3 (NHE3) ELISA detection kit, mouse short-chain fatty acid (SCFA) ELISA detection kit, 4% paraformaldehyde. 4% paraformaldehyde,Absolute ethanol (AR grade), xylene (AR grade), hematoxylin stain, eosin stain, hydrochloric acid (AR grade), neutral gum.

### 2.2 Laboratory animals

ICR mice: 50, 25 male and 25 females; Weight: 20 ± 2 g; From the Laboratory Animal Center of Yunnan University.

### 2.3 Preparation of compound Chinese medicine preparation F1 and senna

F1: Baishu 20 g, Poria 12 g, Codonopsis 15 g, Dried Ginger 20 g, Yam 20 g, Chuanxiong 15 g, Knotweed 10 g, Peran 10 g, Zeeland 10 g, Coptis 6 g, Divine Comedy 12 g. The ingredients were mixed and crushed in the specified proportions. The resulting mixture was then passed through a 120 mesh sieve and sealed for storage. For preparation, 200 g of the powder was soaked in 2000 mL of warm water for 2 h and boiled for 1 h. The chemical solution was filtered using 8 layers of gauze, and the resulting filtrate was evaporated and concentrated at 60°C under reduced pressure until the volume reached 400 mL. This crude drug solution had a concentration of 500 mg/mL and was stored at 4°C for future use.

Senna aqueous solution was prepared as follows: 30 g of senna was soaked in 100 mL of warm water for 1 h and then boiled for 15 min. After filtration through 8 layers of gauze, the solution was centrifuged at 5000 r/min for 5 min. The supernatant volume was adjusted to 50 mL, resulting in a concentration of 600 mg/mL. The solution was stored at 4°C for future use.

### 2.4 Establishment and treatment of SDD mouse model

After adapting 50 mice for 5 days, they were randomly divided into two groups: the experimental group and the control group. Both groups were managed with the same feeding protocol. In the experimental group, each mouse was orally administered 0.5 mL of senna water extract twice a day for 7 days, following a 5-h fasting period before each administration. The control group received the same volume of normal saline. Throughout this period, the clinical manifestations of the mice were observed, and the diarrhea rate and diarrhea index were calculated as the primary indicators for evaluating the diarrhea models.

After successful modeling, three mice were randomly chosen from the experimental group for sampling and dissection (recorded as sdd); Subsequently, twenty mice were selected for treatment with F1 (denoted as f1). Each mouse received a dosage of 1 mg/g Chinese medicine solution through oral gavage, based on their body weight. The administration was conducted once in the morning and once in the evening, continuously for five days. The control group (recorded as nc), received normal feeding without any additional treatment.

### 2.5 Sample collection and processing

After successful modeling and drug treatment, three mice were randomly selected from each group. One milliliter of blood was collected from the heart using ordinary blood collection tubes. The collected blood was allowed to stand for 4 h and then centrifuged at 4000 r/min for 8 min to separate the serum. The serum was transferred to a 2 mL centrifuge tube and stored at −20°C. Simultaneously, the mice were dissected after their necks were broken, and the small intestinal tissues and spleen were collected and stored in 4% paraformaldehyde at 4°C. The contents of the small intestine were also collected and stored by freezing them with liquid nitrogen.

### 2.6 Clinical status monitoring of mice

The study recorded the appearance of coat, behavioral activity, mental state, appetite (feed consumption), fecal morphology, weight change, and death of mice. Additionally, the number of effective treatments, cases of cure, deaths, and recurrences were recorded for each treatment group. The effective rate, cure rate, mortality rate, and recurrence rate were then calculated for each group.

### 2.7 Blood biochemical detection of mice

The levels of alanine aminotransferase (ALT), aspartate aminotransferase (AST), serum total protein (TP), albumin (ALB), urea (UREA), creatinine (CREA), and other components were measured using a veterinary automatic blood biochemistry analyzer.

### 2.8 Determination of spleen and intestinal injury repair in mice

The spleen and intestinal tissues were dehydrated, trimmed, embedded, sectioned, stained, and sealed after paraformaldehyde fixation. They were then subjected to microscopic examination. The BA210 digital trinocular microcamera system was used to capture slice images. Each section was observed at low magnification to identify any tissue lesions. Specific lesions were further examined using 100x and 400x pictures. The extent of the lesion was determined using a four-stage grading approach. Additionally, the height of intestinal villi, crypt depth, and muscle thickness were measured using the line tool.

### 2.9 NHE3 and SCFAs expression

The expression of serum NHE3 and SCFAs was determined using the bianti-antibody sandwich ELISA method by creating a linear regression model based on the creating of the standarded curve.

### 2.10 Detection of intestinal microbial diversity in mice

The intestinal contents of mice were frozen using liquid nitrogen and then sent to Beijing Baimeike Biotechnology Co., Ltd. for next-generation sequencing of the 16S rRNA V3-V4 region to analyze the intestinal bacteria.

### 2.11 Data statistics and analysis

Preliminary statistical collation of data was performed using Excel 2019, followed by statistical analysis using GraphPad Primim 8.0.2. The results were represented as mean ± standard error. A significant difference was denoted by *p* < 0.05, indicated by “*,” while a greater difference was denoted by *p* < 0.01, represented by “**.” Gut microbiome analysis was conducted using BMKCloud.^1^

## 3 Results

### 3.1 Treatment results

Table 1 presents the number of treatments and cures, indicating that the effective rate reaches 95% after 5 days of treatment and the cure rate reaches 95% after 6 days. Additionally, the recurrence rate and mortality rate during this period are low.

**Table 1:** Statistics of treatment results of spleen deficiency and diarrhea in mice.

	Significant number	Efficient	Number of cures	Cure rate	Number of deaths	Mortality	Number of recurrences	Relapse rate
1d	8	40%	0	0	0	0	0	0
2d	11	55%	7	35%	0	0	0	0
3d	15	75%	12	60%	1	5%	0	0
4d	17	85%	15	75%	0	0	1	11.11%
5d	19	95%	18	90%	0	0	0	0
6d	19	95%	19	95%	0	0	0	0
7d	19	95%	19	95%	0	0	0	0

### 3.2 Blood biochemical results

**Figure 1:**
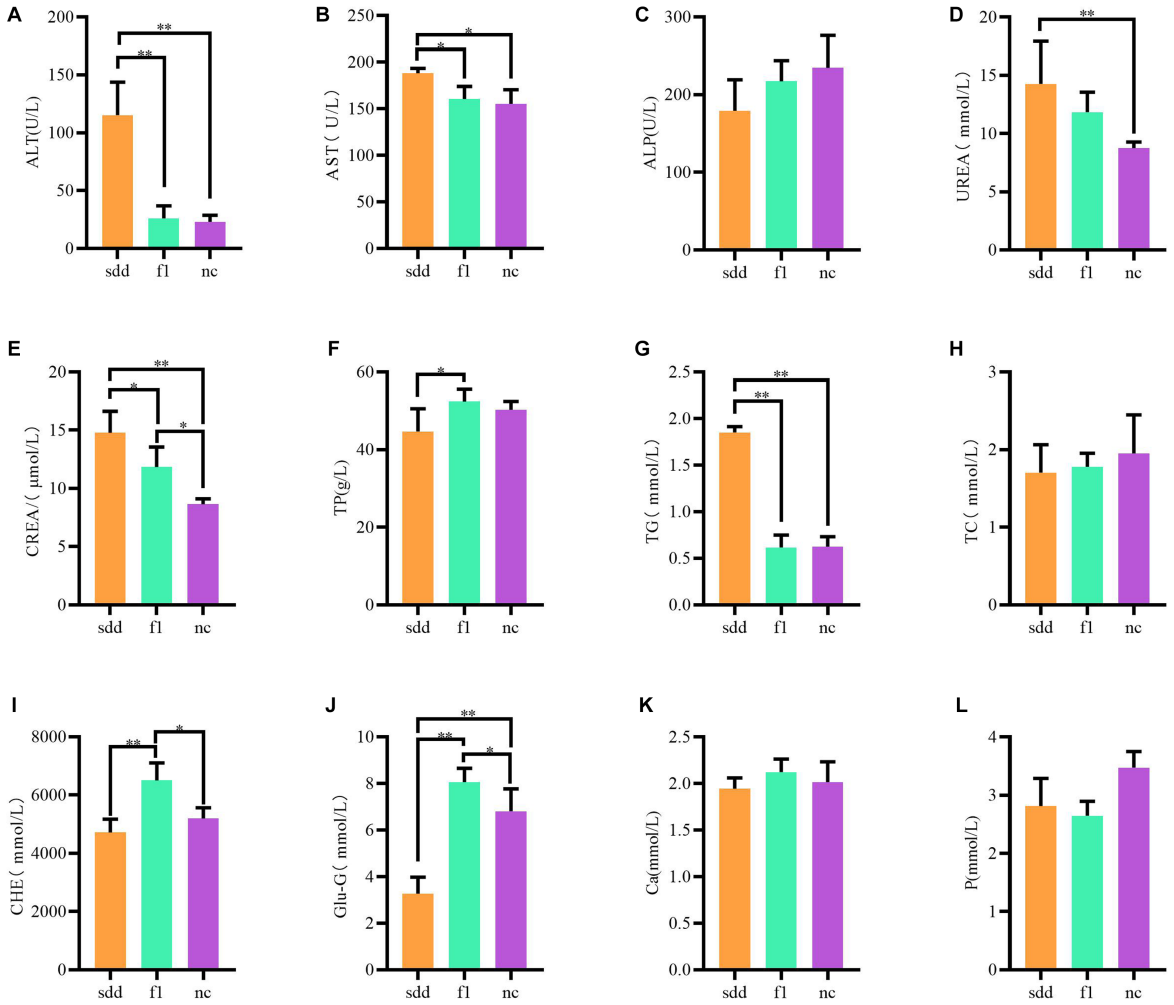
Analysis of differences in blood biochemical tests in mice. **(A–L)** Represents a histogram of 12 different blood biochemical indicators; All abscissa indicates grouping information (orange for sdd group, green for fl group, purple for nc group); Ordinates represent numeric values.

The blood biochemistry of mice in each treatment group was analyzed using a veterinary automatic blood biochemistry instrument, and the results were statistically analyzed (Table 2 and Figure 1). The levels of ALT, TG, UREA, and crea-S in the sdd group were significantly higher than those in the nc group (*p* < 0.01). Additionally, the levels of AST were significantly higher than those in the nc group (*p* < 0.05), while the Glu-G levels were significantly lower (*p* < 0.01). Following F1 treatment, the liver and kidney function indexes of mice showed a recovery, with the exception of slightly higher levels of CREA-S, CHE, and Glu compared to the nc group. However, there was no significant difference in other indexes (*p* > 0.05).

**Table 2:** Results of blood biochemical test results of mice in each group of spleen deficiency and diarrhea.

Project		sdd	F1	nc
ALT	U/L	115.2000 ± 14.2700	26.08 ± 5.4610	23.0800 ± 2.8420
AST	U/L	188.3000 ± 2.6360	160.6000 ± 6.7540	155.3000 ± 7.6210
ALP	U/L	179.2000 ± 19.9500	217.0000 ± 11.9100	234.3000 ± 18.8800
UREA	mmol/L	14.2700 ± 1.8300	11.8400 ± 0.7727	8.7630 ± 0.2089
CREA-S	μmol/L	14.7700 ± 0.9228	11.8400 ± 0.7727	8.6440 ± 0.2100
TPII	g/L	44.6900 ± 2.9180	52.3600 ± 1.4360	50.2800 ± 0.9495
TG	mmol/L	1.8500 ± 0.0319	0.6160 ± 0.0594	0.6260 ± 0.0474
TC	mmol/L	1.7030 ± 0.1803	1.7780 ± 0.0788	1.9520 ± 0.2225
CHE	U/L	4,716 ± 227.3000	6,504 ± 301.1000	5,194 ± 185.7000
Glu-G	mmol/L	3.2700 ± 0.3536	8.066 ± 0.2623	6.8100 ± 0.4811
Ca	mmol/L	1.9450 ± 0.0578	2.1240 ± 0.0628	2.0150 ± 0.1550
P	mmol/L	2.8150 ± 0.2374	2.6500 ± 0.1102	3.4750 ± 0.1950

### 3.3 Histoscopy observation and pathology of lesions

#### 3.3.1 Repair of spleen damage

**Figure 2:**
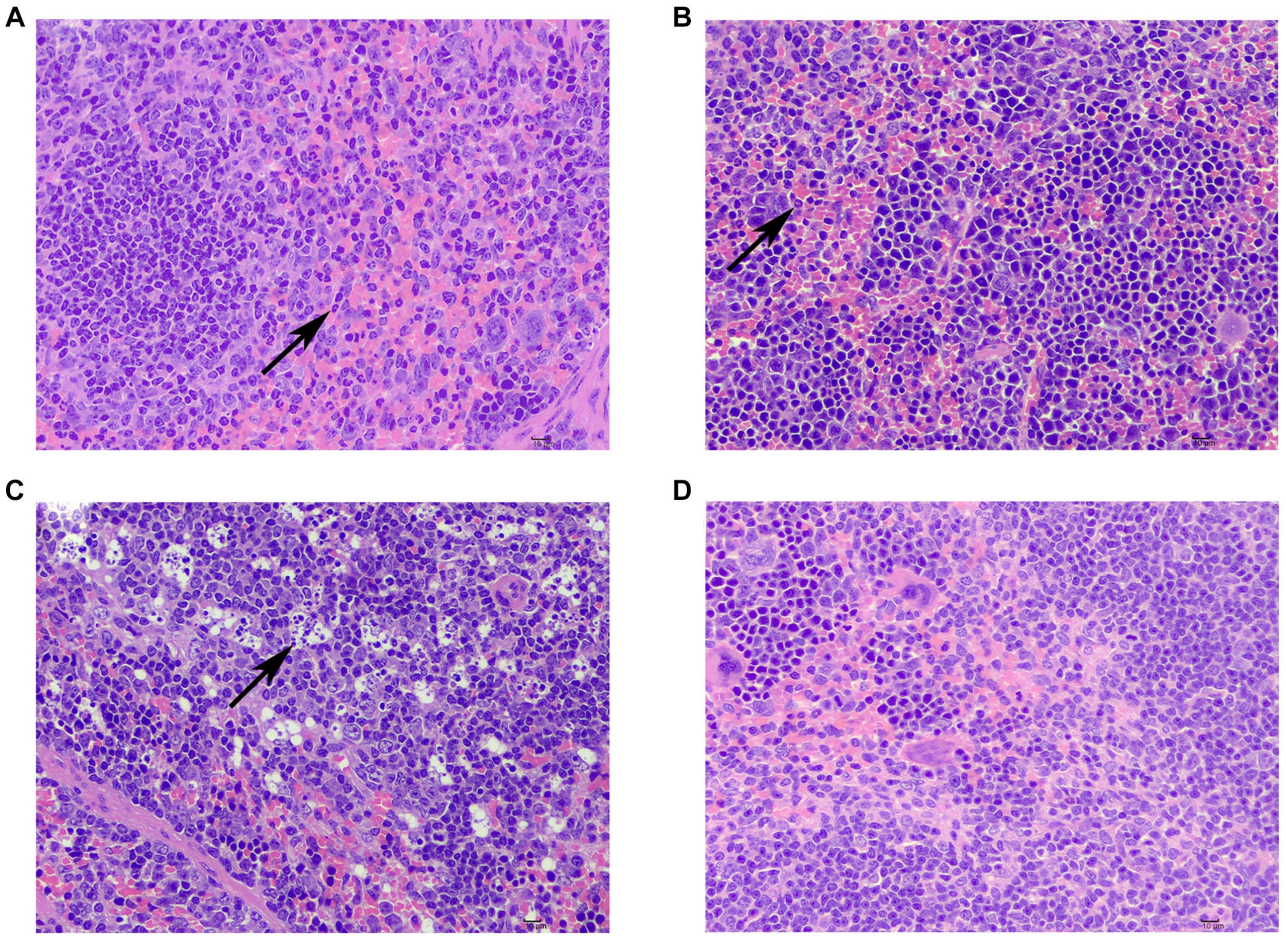
Spleen injury and repair in mice. **(A)** Lymphocyte necrosis in the splenic nodule of albilopulp (↑); **(B)** neutrophilia within the white pulp (↑); **(C)** increased number of naïve granulocytes in tissues (↑); **(D)** No obvious abnormalities were seen.

One patient in the sdd group exhibited lymphocyte necrosis in the splenic nodule of albidon, along with cytosolic lysis of necrotic cells. Additionally, there was solid shrinkage and disintegration of nuclei, resulting in the phenomenon commonly referred to as ‘full of stars’ (Figure 2A). In another case, there was an increase in cell components in the pulp, as well as the presence of lobulated nuclei or rod-shaped nuclei neutrophils (Figure 2B). Mild extramedullary hematopoiesis was observed in one case, with an increased number of naïve granulocytes in the tissue (Figure 2C). Following F1 treatment, the spleen tissue membrane remained intact, and there was no proliferation of the membrane or splenic trabecular fibrous connective tissue. The demarcation between white pulp and red pulp was clear, and there was no hyperplasia or atrophy observed in the white pulp splenic nodule, with no significant decrease in the number of cells. No obvious proliferation or decrease of various cellular components was noted in the red medullary, and the spleen and blood sinuses formed a network without any signs of congestion or inflammatory cell infiltration.

#### 3.3.2 Damage to the intestines

**Figure 3:**
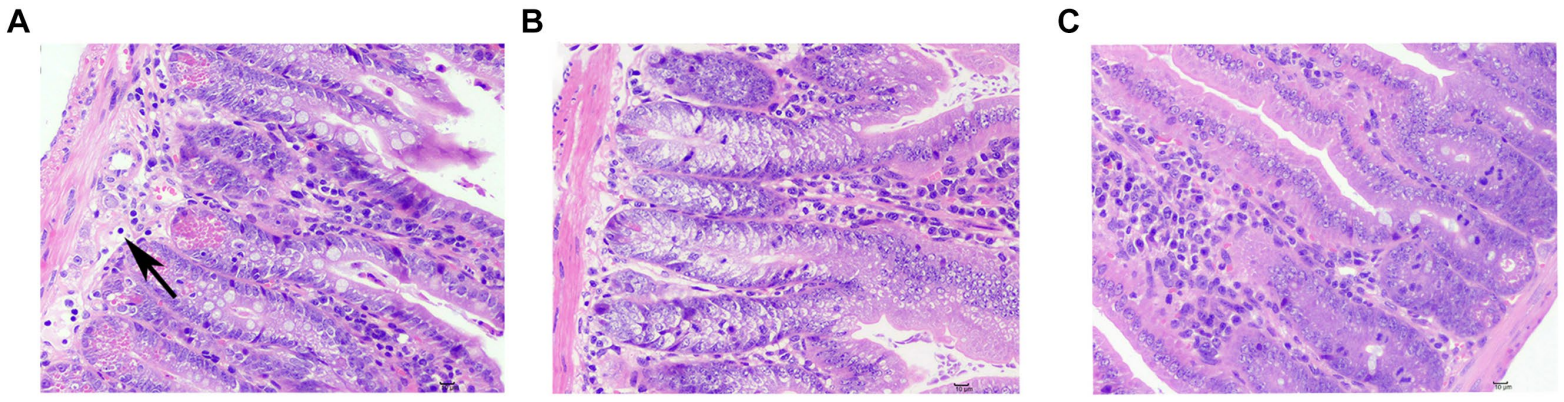
Pathological slides of small bowel injuries. **(A)** Typical lesions in the sdd group, inflammatory cell infiltration (↑). **(B)** f1 group showed no obvious pathological changes, and one was randomly selected as a representative picture. **(C)** No obvious pathological changes were seen in the nc group, and one was randomly selected as a representative picture.

In one case from the sdd group, a minor amount of inflammatory cell infiltration was observed, primarily consisting of lymphocytes with oval nuclei. There was no notable inflammatory cell infiltration or hyperplasia in the outer membrane layer. No other significant pathological changes were observed after treatment (see Figure 3).

### 3.4 Intestinal tissue measurement

**Figure 4:**
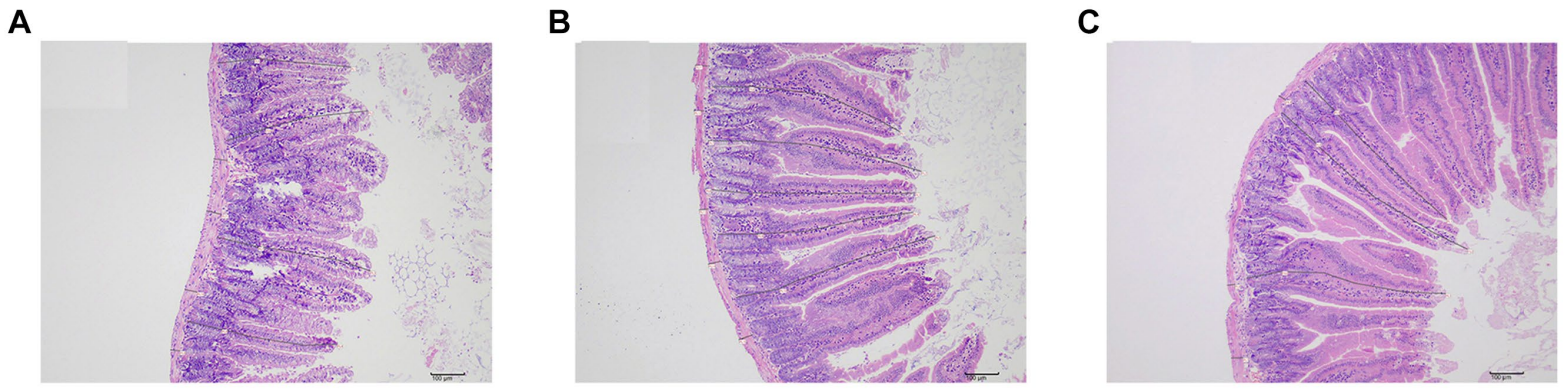
Small intestinal tissue measurements. **(A)** Representative figure of intestinal villi height, crypt depth and muscular thickness measurement in sdd group. **(B)** Representative figure of intestinal villi height, crypt depth and muscular thickness measurement in fl group. **(C)** Representative figure of intestinal villi height, crypt depth and muscular thickness measurement in nc group.

After measurement (Table 3 and Figure 4), the height of intestinal villi was found to be significantly lower in the sdd group compared to both the f1 group and the nc group (*p* < 0.01). However, there was no significant difference in the height of intestinal villi between the f1 group and the nc group (*p* > 0.05). The crypt depth in the sdd group was significantly higher than that in the f1 group and the nc group (*p* < 0.01), while there was no significant difference in crypt depth between the F1 group and the nc group (*p* > 0.05). Additionally, the muscular layer in the sdd group was significantly higher than that in the nc group (*p* < 0.01) and also higher than that in the F1 group, although the difference between the f1 group and the nc group was not significant (*p* > 0.05).

**Table 3:** Small intestinal tissue measurement data of mice with spleen deficiency and diarrhea.

Project		sdd	F1	nc
Intestinal villi height	μm	250.9 ± 14.09	438.3 ± 11.74	482.0 ± 7.237
Crypt depth	μm	116.5 ± 3.580	88.25 ± 3.669	88.50 ± 3.190
Muscular layer thickness	μm	34.82 ± 2.182	27.05 ± 2.060	26.20 ± 1.453

### 3.5 NHE3 and SCFAs expression

**Figure 5:**
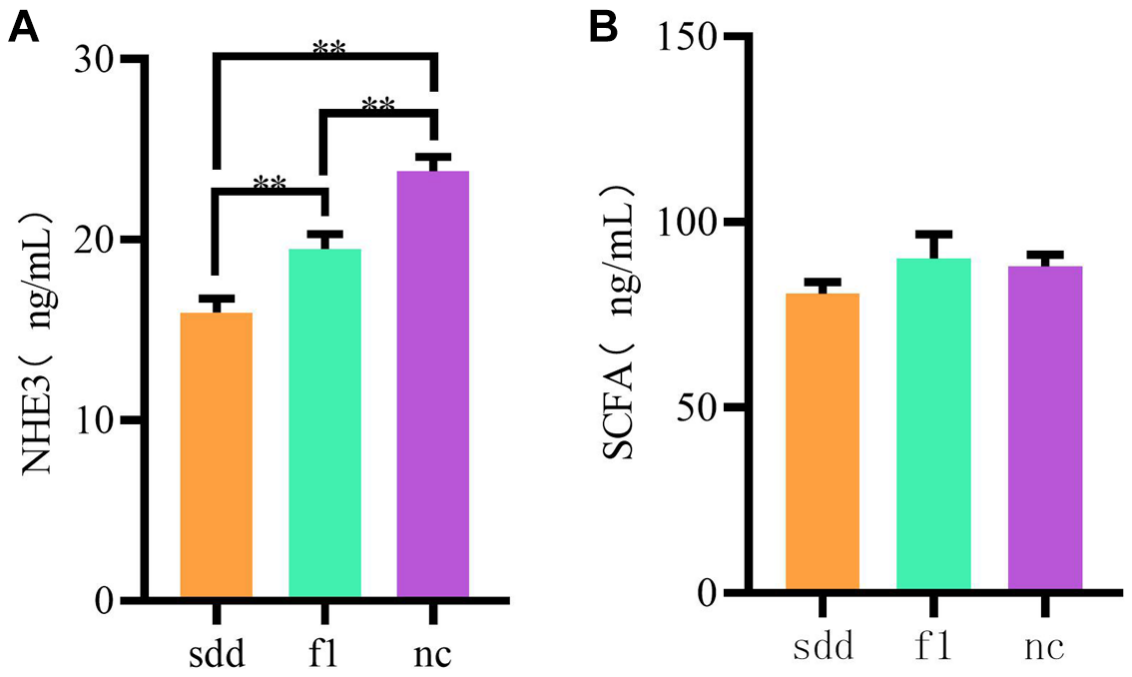
Mouse serum NHE3 and SCFAs expression. **(A)** Is a histogram of serum NHE3 expression, and **(B)** is a histogram of serum SCFA expression. The abscissa coordinates are all grouping information (orange indicates sdd group, green indicates fl group, purple nc indicates blank control,); The ordinate represents the expressed numerical value.

The serum NHE3 content in the sdd group was significantly lower than that in the f1 and nc groups (*p* < 0.01), and the f1 group was significantly lower than that in the nc group (*p* < 0.01) (Table 4 and Figure 5A).

**Table 4:** Mouse NHE3 /detection results.

		sdd	f1	nc
NHE3	ng/mL	15.95 ± 0.3955	19.48 ± 0.4236	23.80 ± 0.4512
SCFAs	ng/mL	80.74 ± 3.085	90.15 ± 6.520	88.10 ± 3.114

There was no significant difference in serum SCFAs content between the three groups (*p* > 0.05), but the f1 content was the highest (Table 4 and Figure 5B).

### 3.6 Intestinal flora sequencing results

#### 3.6.1 Sequencing data quality assessment

A total of 719,228 pairs of Reads were obtained from the three sequencing groups. After double-ended Reads quality control and splicing, a total of 714,143 CleanReads were generated (Table 5). There were no significant differences observed in RawReads, CleanReads, DenoisedReads, MergedReads, Non-chimericReads, and ASVs between the three groups (*p* > 0.05).

**Table 5:** Sequencing data quality.

Project	Unit	Outcome		
		sdd	f1	nc
RawReads	Strip	79,878 ± 113.5	79,939 ± 52.92	79,925 ± 70.97
CleanReads	Strip	79,310 ± 106.7	79,373 ± 61.61	79,365 ± 62.27
DenoisedReads	Strip	77,977 ± 326.0	77,297 ± 405.5	76,600 ± 1,061
MergedReads	Strip	76,210 ± 1,018	74,733 ± 837.3	73,250 ± 2,265
Non-chimericReads	Strip	68,534 ± 2079	66,639 ± 530.0	62,802 ± 2,989
OTU_Num		436.0 ± 184.5	465.0 ± 105.5	379.7 ± 53.82
Seqs_Num		68,521 ± 2074	66,624 ± 529.7	62,771 ± 3,010

#### 3.6.2 Species distribution analysis

**Figure 6:**
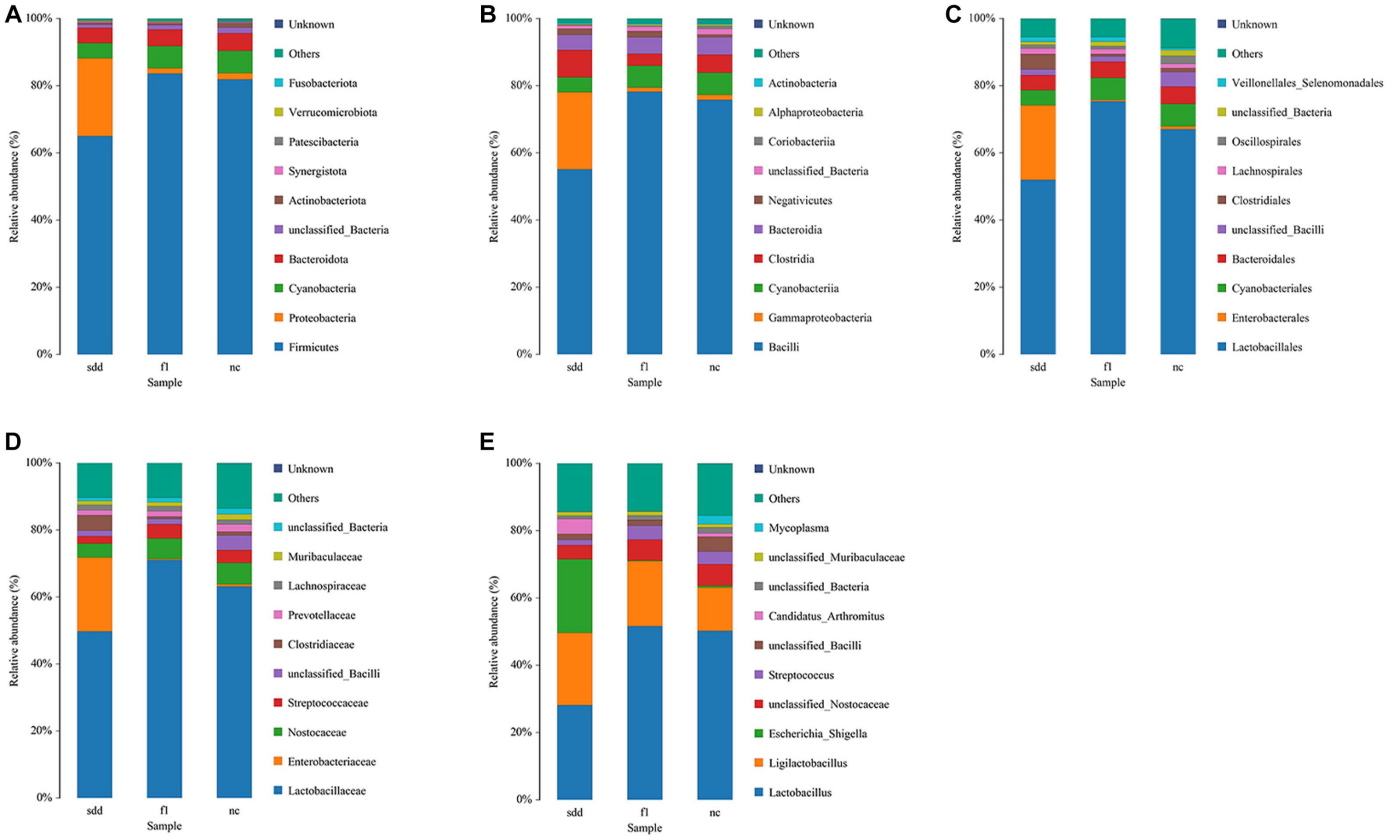
Distribution of intestinal microbial community composition in mice. **(A–E)** Represent the histogram of the structure of the intestinal colonies of the three groups of mice from the phylum to the genus level. The abscissa is grouped information; The ordinate represents the composition percentage; Different color legends represent different flora information.

At the phylum level, the dominant community composition of the intestinal colony in the three groups of mice was Firmicutes. sdd showed approximately 20% less Firmicutes and approximately 20% more Proteobacteria compared to f1 and nc (Figure 6A).

At the class level, the dominant community composition of the intestinal flora in the three groups of mice was Bacilli. sdd showed approximately 20% less Bacilli than f1 and nc, while Gammaproteobacteria increased by approximately 20% (Figure 6B).

At the order level, the dominant community composition of the intestinal flora in the three groups of mice was Lactobacillales. sdd exhibited a decrease of approximately 16% in Lactobacillales and approximately 22% in Enterobacterales compared to nc (Figure 6C).

At the family level, the dominant community composition of the intestinal flora in the three groups of mice was Lactobacillaceae. sdd showed a decrease of approximately 13% in Lactobacillaceae and approximately 22% in Enterobacteriaceae compared to nc (Figure 6D).

At the genus level, the intestinal colony community composition of the three groups of mice was predominantly composed of Lactobacillus. However, there were variations in the species composition and proportion among the groups. In comparison to the nc group, the sdd group exhibited a decrease of approximately 22% in lactobacillaceae, an increase of around 21% in Escherichia_Shigella, and a rise of about 9% in Ligilactobacillus (Figure 6E).

#### 3.6.3 Alpha diversity analysis

**Figure 7:**
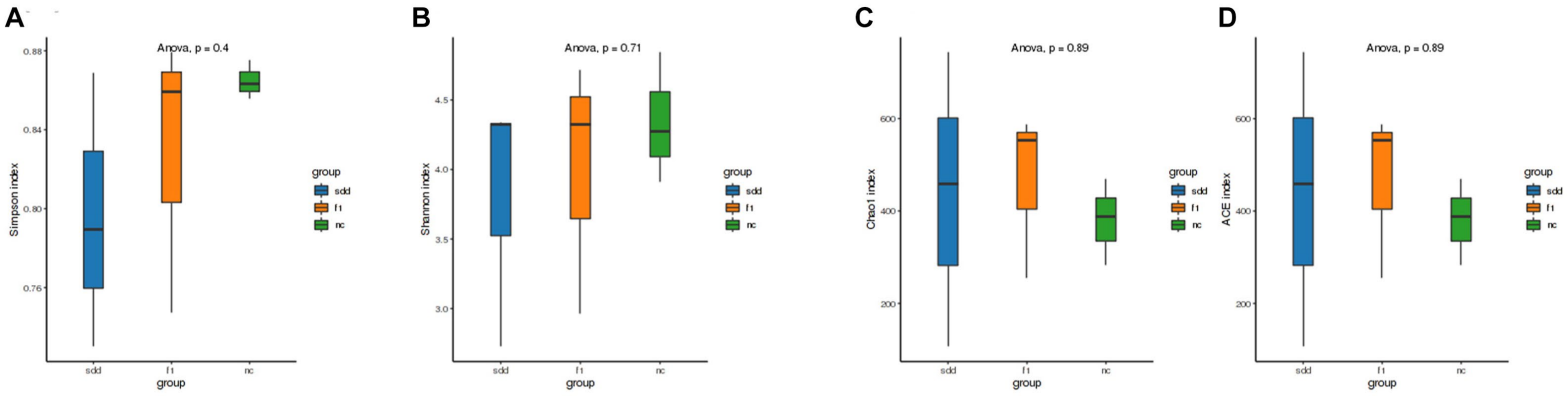
Analysis of alpha diversity of mouse intestinal flora. **(A–D)** Represents the four index difference analysis charts of Simpson, Shannon, Chaol, and Ace in three groups. The abscissa is grouped information; Ordinates represent numeric values; Different color legends represent different groups.

The sample Alpha diversity index was evaluated using the QIIME2 2020.6 software (Figure 7). There were no significant differences (*p* > 0.05) observed between the six indexes of Ace, Chao1, Simpson, Shannon, Coverage, and PD_whole_tree. However, the Ace, Chao1, and PD_whole_tree indices were highest in the f1 group, followed by the sdd group, and lowest in the nc group. Group nc exhibited the largest Simpson and Shannon indices.

## 4 Discussion

The compound traditional Chinese medicine preparation has shown significant effectiveness in treating spleen deficiency and diarrhea in mice. The treatment demonstrated a faster effect, with 40% of mice showing significant improvement after just 1 day of medication. By the fifth day, the cure rate reached 90%, with the exception of one weaker mouse that unfortunately died midway. All the remaining mice were successfully cured after a six-day medication period. The mice with spleen deficiency and diarrhea exhibited much higher levels of serum ALT, AST, TG, UREA, CREA-S, etc., compared to the normal group of mice. In particular, ALT levels were almost five times higher than normal, and Glu levels were significantly lower than those in the control group. These findings indicate that long-term administration of senna not only leads to spleen deficiency and diarrhea in mice but also causes damage to the liver and kidney function. However, after 7 days of treatment with the compound traditional Chinese medicine preparations, the liver and kidney function indexes returned to normal, with no significant difference compared to the normal group, except for slightly higher levels of CREA-S, CHE, and Glu. From the perspective of serum biochemical indexes, the presence of spleen deficiency and diarrhea is accompanied by some degree of liver and kidney function damage. Although structural damage cannot be confirmed without pathological sections and urine sediment examination, the study demonstrates that the compound traditional Chinese medicine preparation not only promotes the recovery of liver and kidney function in mice with spleen deficiency and diarrhea, but also increases serum total protein and blood glucose levels.

The spleen, as the largest secondary immune organ, plays a crucial role in disease development. It is responsible for the transport and localization of lymphocytes in the splenic microenvironment, allowing them to scan antigen-presenting cells (Lewis et al., 2019). Lymphocyte necrosis and disintegration can result in weakened immunity. This compound traditional Chinese medicine has been found to have a reparative effect on spleen damage, thereby enhancing the immune function of mice.

In this study, we observed intestinal paraffin sections and performed HE staining before and after treatment and recovery. We also measured and compared the numerical values of intestinal villi and crypts. These observations provided a more intuitive understanding that the compound Chinese medicine can promote the regeneration of intestinal villi and repair damaged mucosa. However, further research and exploration are needed to determine whether this compound TCM preparation can promote the proliferation and differentiation of intestinal stem cells and tufted mesenchymal cells, facilitate the regeneration of wound-associated epithelial (WAE) cells (Quirós and Nusrat, 2019), or increase the secretion of Hh ligand by pseudo-multilayer intestinal epithelium and the expression of myosin II-related genes. It is important to note that compound traditional Chinese medicine preparations have a complex composition and exhibit multi-action and multi-target characteristics.

Spleen deficiency and diarrhea in mice were found to have significantly lower serum NHE3 levels compared to normal groups. Previous studies have shown that excessive stimulation of bacterial heat-stable enterotoxin STa, along with cGMP accumulation and an increase in protein kinase A, strongly inhibits NHE3. Additionally, certain viral infections causing diarrhea can also inhibit NHE3 protein (Weiglmeier et al., 2010). It has also been research discovered that Jin-PiY-in can reduce the expression of GLP-1, reduce the ubiquitination and phosphorylation of NHE3, regulate the expression of NHE3, at least partly improve ion transport in the intestinal epithelium, and improve the imbalance of electrolyte absorption, thus significantly reducing the diarrhea symptoms (Ma et al., 2023). In this study, the level of NHE3 improved after treatment with compound traditional Chinese medicine preparations, indicating that promoting the expression of NHE3 protein may be one of the mechanisms by which these preparations treat spleen deficiency and diarrhea.

For mice with spleen deficiency and diarrhea, the intestinal flora exhibited significant changes in the abundance of Firmicutes and Proteobacteriaces. Specifically, there was a notable decrease of approximately 22% in the abundance of Lactobacillus, while the abundance of Shigella increased by approximately 21%. Although some changes were observed in other phyla and genus, they were relatively minor in magnitude. These findings provide evidence that spleen deficiency leads to a compromised defense qi, resulting in susceptibility to external pathogens. Consequently, the intestinal micro-ecosystem loses its self-stable state, leading to damage to the intestinal mucosal barrier and immune function disorders. Following treatment with compound traditional Chinese medicine preparations, the abundance of Lactobacillus under the phylum Firmicutes reached 51.6%, while the abundance of Shigella under the phylum Proteobacteria decreased to 0.2%. Overall, the flora structure and composition were nearly identical to those of the normal groups.The study indicated that the compound traditional Chinese medicine preparation effectively increased the abundance of intestinal firmicutes and Lactobacillus in mice with spleen deficiency and diarrhea. This, in turn, led to an increase in the abundance of beneficial bacteria while inhibiting the growth and reproduction of pathogenic bacteria like Shigella. The compound also demonstrated the ability to promote the restoration of homeostasis in the intestinal flora and repair the biological barrier of the intestinal mucosa.

In this experiment, the serum SCFAs content of mice was quantitatively analyzed by double anti-sandwich enzyme-linked immunoassay, and the dysbacteriosis of the mice was caused by spleen deficiency and diarrhea, and the SCFAs level was lower than that of the blank control group. Decreased biosynthesis of SCFAs affects the barrier function of the intestine and increases the translocation of intestinal wall endotoxins, thereby triggering chronic inflammation and disruption of glucose and lipid metabolism in the body (Xiong et al., 2022). The SCFAs of mice cured by the compound TCM preparation were slightly higher than those in the control group, indicating that the compound TCM preparation had a certain promoting effect on SCFAs, but only the total SCFAs were measured and analyzed, and the specific components were not determined, resulting in no significant difference. Due to the low concentration and lack of standardization of SCFAs, which are difficult to measure clinically, veterinary clinical research on SCFAs remains a challenge.

## 5 Conclusion

The compound traditional Chinese medicine preparation F1 is involved in the treatment of spleen deficiency and diarrhea by regulating the body’s sodium and hydrogen exchange proteins, intestinal mucosa, intestinal flora, and metabolites.

## Data availability statement

The original contributions presented in the study are publicly available. This data can be found at: https://figshare.com/10.6084/m9.figshare.24460048.

## Ethics statement

The animal study was approved by Ethical Review Committee of Laboratory Animal Welfare of Yunnan University. The study was conducted in accordance with the local legislation and institutional requirements.

## Author contributions

FD, KW, and GL Conceptualization, Methodology, Visualization, Validation, Analysis, and Writing - draft. ZY, FY, YS, GD, WS, KZ, and JH Writing - review & revision. All authors contributed to the article and approved the submitted version.

## Footnotes

^1^www.biocloud.net
